# Obituary

**Published:** 1910-03-12

**Authors:** 


### Obituary.

The death by drowning is announced of Dr. George Waring, medical practitioner of Ulster. He became a licentiate and licentiate in midwifery of King's and Queen's Colleges of Physicians and Surgeons, Ireland, in 1888.

The death occurred suddenly, at Snaith, near Goole, of Dr. Archibald Auld, one of the best known medical men in the district. Dr. Auld, who graduated M.B., C.M. at Glasgow in 1888, held many public appointments, chief of which were those of public vaccinator and medical officer of health for Snaith, Rawcliffe, and district.

The death is reported at Lichfield of Dr. James Clark, M.D., at the age of seventy-five. He became an M.D. of Aberdeen in 1864 and an F.R.C.S. of Edinburgh in 1870. He was President of the Staffordshire British Medical Association, and had been consulting surgeon to Hammerwich and District Cottage and Walsall Hospitals, medical referee to the National Hospital for Consumption, Ventnor, M.O.H. for Lichfield. He had also retired from the 2nd V.B. South Staffordshire Regiment, of which he was surgeon-lieutenant-colonel.

Dr. John W. Taylor, late senior surgeon to the Birmingham and Midland Hospital for Women, and Professor of Gynæcology in the University of Birmingham, has died at Birmingham. He was M.D. of Brussels (1877), M.Sc. of Birmingham (1901), and F.R.C.S. (1877). He studied at Charing Cross Hospital, and among other appointments held that of consulting surgeon to the Wolverhampton Hospital for Women, to the Birmingham and Midland Skin and Lock Hospital, besides resident surgical and medical posts at Charing Cross. He was the writer of numerous articles and monographs on gynæcological subjects.

The death is announced at Dublin of Dr. Edward Percival Wright, F.L.S., late Professor of Botany in Trinity College, at the age of 76. Educated at Trinity College, he took his B.A. in 1857, his M.B. in 1858, and his M.D. in 1862. He gained a great reputation as a botanist, and was a member of many botanical and zoological societies on the continent and at home. He was a well-known figure in Dublin, and acted in 1899 as Secretary of the Royal Irish Academy. In addition to many publications, which were well known among botanists, he was joint-author with T. H. Huxley of a memoir on fossil vertebrates.

